# Klinische Ethikberatung: Ethik geht uns alle an!

**DOI:** 10.1007/s00508-025-02658-9

**Published:** 2025-11-28

**Authors:** Eva Katharina Masel, Lorenz Faihs, Sabine Pleschberger, Jacob Roth, Bruno K. Podesser, Helga Dier, Joachim Widder, Barbara Friesenecker, Stefan Dinges, Klara Doppler, Maria Kletečka-Pulker, Jan Schildmann, Christiane Druml, Eva Schaden

**Affiliations:** 1 Division of Palliative Medicine, Department of Medicine I, Medical University of Vienna, Vienna, Österreich; 2 Herz Jesu Krankenhaus, Vienna, Österreich; 3 Ludwig Boltzmann Institute for Experimental and Clinical Traumatology, Wien, Österreich; 4 Center for Public Health, Medical University of Vienna, Wien, Österreich; 5 Center for Biomedical Research and Translational Surgery, Medical University of Vienna, Wien, Österreich; 6 Clinical Department of Cardiac Surgery, University Hospital St. Pölten, Karl Landsteiner University St. Pölten, St. Pölten, Österreich; 7 Clinical Department of Anesthesia and Intensive Care Medicine, University Hospital St. Pölten, Karl Landsteiner University St. Pölten, St. Pölten, Österreich; 8 Department of Radiation Oncology, Comprehensive Cancer Center, Medical University of Vienna, Wien, Österreich; 9 Department of Anesthesia and General Intensive Care, Medical University of Innsbruck, Innsbruck, Österreich; 10 Institute for Ethics and Law in Medicine, University of Vienna, Wien, Österreich; 11 Ludwig Boltzmann Institute Digital Health and Patient Safety, Wien, Österreich; 12 Institute for History and Ethics of Medicine, Martin Luther University of Halle, Wittenberg, Schweiz; 13 UNESCO Chair on Bioethics, Ethics, Collections and History of Medicine, Medical University of Vienna, Wien, Österreich; 14 Department of Anaesthesia, Intensive Care Medicine and Pain Medicine, Medical University of Vienna, Wien, Österreich

**Keywords:** Klinische Entscheidungsfindung, Kommunikation, Ethik, Interdisziplinäre Forschung, Patientenrechte, Clinical Decision Making, Communication, Ethics, Interdisciplinary Research, Patient Rights

## Abstract

Die Klinische Ethikberatung (KEB) unterstützt Behandlungsteams in komplexen Entscheidungssituationen, indem sie die Autonomie der Patient:innen, die interprofessionelle Zusammenarbeit und die rechtlichen Rahmenbedingungen in den Mittelpunkt stellt. Mit der Einführung der KEB am Allgemeinen Krankenhaus Wien und an der Medizinischen Universität Wien wurde ein strukturiertes Angebot geschaffen, das ethische Fragestellungen systematisch in den klinischen Alltag integriert. Neben der Durchführung von Ethikfallberatungen umfasst die Initiative Fortbildungsangebote, die Entwicklung von Dokumentationsunterlagen zur Therapiezielplanung und die Einbindung in Curricula sowie in die ärztliche Basisausbildung. Das Ziel besteht darin, die Entscheidungskompetenz der Teams zu stärken, ethische Prinzipien sichtbar zu machen und die Qualität der patientenorientierten Versorgung langfristig zu steigern. Das Supplement stellt Hintergründe, Implementierungsschritte und Erfahrungen vor und zeigt praxisnahe Ansätze zur nachhaltigen Verankerung klinischer Ethik in einem zunehmend komplexen Gesundheitswesen auf.

## Abstract (en)

Clinical Ethics Consultation (CEC) provides support to treatment teams in complex decision-making situations, focusing on patient autonomy, interprofessional collaboration and the legal framework. Following the introduction of CEC at the Vienna General Hospital and the Medical University of Vienna, a structured service has been established that systematically integrates ethical issues into everyday clinical practice. As well as conducting ethics case consultations, the initiative includes training programs, development of documentation tools for therapy goal planning and expansion of curricula and basic medical education. The aim is to strengthen teams’ decision-making competence, to make ethical principles visible, and to sustainably increase the quality of patient-centered care. This supplement presents the background, implementation steps and experiences of the initiative, and illustrates practical approaches to embedding clinical ethics within an increasingly complex healthcare system in a sustainable way.

## Können Sie Ethik? Entscheiden mit Verantwortung: Ethik im klinischen Alltag (Eva Katharina Masel, Eva Schaden)

### Ursprünge der Klinischen Ethikberatung

Klinische Ethikberatung (KEB) hat sich in den letzten fünf Jahrzehnten als strukturierte Einrichtung im Gesundheitssystem etabliert. Ziel der KEB ist es, bei ethischen Fragestellungen, Meinungsverschiedenheiten oder Konflikten das Behandlungsteam bei der Entscheidungsfindung – durch Information, Moderation und eine vermittelnde Haltung, die das Verständnis für alle beteiligten Perspektiven fördert – zu unterstützen. Neutralität bedeutet in diesem Zusammenhang nicht Wertfreiheit oder emotionale Distanz, sondern empathische Offenheit gegenüber unterschiedlichen Sichtweisen [1].

Die Anfänge dieser Entwicklung lassen sich bis in die 1960er-Jahre zurückverfolgen, als die Verteilung knapper Ressourcen – insbesondere die neu eingeführte Hämodialyse – ethische Entscheidungen über Leben und Tod erforderte. In dieser frühen Phase lag der Fokus stark auf konkreten Entscheidungsprozessen, was sich beispielsweise in der Überschrift eines LIFE-Magazin-Artikels aus dem Jahr 1962 widerspiegelt, in dem über eines der ersten klinischen Ethikkomitees berichtet wurde: „They decide who lives, who dies“.

Der medizinische Fortschritt in Bereichen wie der Intensiv- und Notfallmedizin und der Transplantationsmedizin, die Technisierung sowie gesellschaftliche Debatten zu Themen wie Hirntod, Schwangerschaftsabbruch und Pränataldiagnostik haben die Notwendigkeit ethischer Reflexion verstärkt. Die Verantwortung gegenüber dem einzelnen Menschen rückt stärker in den Vordergrund, sodass die Bedeutung expliziter ethischer Expertise und gemeinsamer Reflexion – so wie in der medizinischen Forschung bereits selbstverständlich – auch in der klinischen Praxis zunehmend anerkannt wird.

### Klinische Ethikberatung im klinischen Alltag

Im klinischen Alltag werden Mitarbeiter:innen häufig mit herausfordernden ethischen Fragestellungen konfrontiert, insbesondere im Kontext komplexer Behandlungsverläufe oder bei divergierenden Wertvorstellungen innerhalb des therapeutischen Teams [2, 3]. Die KEB verfolgt das Ziel, durch einen strukturierten Beratungsprozess Reflexionsräume für alle Mitarbeitenden, die an der Behandlung betroffener Patient:innen beteiligt sind, als auch (selten) für die Patient:innen und ihre An- und Zugehörigen selbst, zu eröffnen, in denen Werte- und Zielkonflikte thematisiert, Therapieziele fachlich fundiert und ethisch begründet diskutiert und entsprechende Behandlungsentscheidungen und Therapiezieländerungen begleitet werden können [4].

Ein Ethikberatungsteam setzt sich in der Regel multiprofessionell zusammen, um eine möglichst große Perspektivenvielfalt in der ethischen Bewertung zu gewährleisten. Dazu gehören meist medizinisches Fachpersonal wie Ärzt:innen und Pflegepersonen, die die klinische Sicht vertreten, sowie Ethikexpert:innen mit einem Hintergrund in Philosophie, Theologie oder Medizinethik. Ergänzt wird das Team häufig durch juristische Expert:innen. Die Ethikfallberatung ist nach dem Prinzip des „Runden Tisches“ organisiert, was eine partizipative Einbindung aller betroffenen Akteur:innen unter Wahrung ihrer jeweiligen Rechte und Verantwortlichkeiten auf Augenhöhe sicherstellt.

Zentrales Ergebnis des Beratungsprozesses ist eine ethisch fundierte, multiperspektivische Einschätzung der vorliegenden Situation sowie eine entsprechende Empfehlung an das behandelnde Team [5]. Diese Empfehlung wird in die Patient:innenakte aufgenommen, wobei die Letztverantwortung für die Umsetzung der vorgeschlagenen Maßnahmen beim behandelnden Team verbleibt.

### Ethische Erwägungen und Entscheidungsfindung bei der Begrenzung kurativer Therapien

Die Entscheidung, bei Patient:innen eine kurative Therapie zu beenden und zu einer ausschließlich palliativen Versorgung überzugehen, basiert auf der Achtung der Menschenwürde und dem Anspruch auf eine verantwortungsvolle und der Situation angemessene Behandlung. Kurative Maßnahmen in aussichtslosen Situationen können das Leiden verlängern und ein Abschiednehmen verhindern.

In solchen Fällen steht die Linderung von Symptomen und das Ermöglichen eines Sterbens in Würde in Form von „Sterben zulassen“ im Vordergrund [6]. Eine verantwortungsvolle Therapie benennt klar das Therapieziel, orientiert sich am Willen bzw. mutmaßlichen Willen der Patient:innen und wendet angemessene Mittel an. Daraus kann sich die bewusste Entscheidung ergeben, auf lebensverlängernde Maßnahmen zu verzichten.

Diese Entscheidung wird vom verantwortlichen ärztlichen Personal in Absprache mit dem therapeutischen Team getroffen. Sie wird dokumentiert und (falls möglich) mit den Patient:innen sowie ihren An- und Zugehörigen offen kommuniziert.

Dabei spielt die psychische Entlastung des Personals eine wesentliche Rolle [7]. Die Ursachen für „moral distress“ bzw. „moral injury“ im klinischen Alltag können vielfältig sein und resultieren häufig aus einer Kombination von organisatorischen, kommunikativen und emotionalen Belastungen. Unklare Versorgungsziele und inkonsistente Behandlungspläne führen zu Unsicherheiten und inneren Konflikten bei den Mitarbeitenden. Besonders belastend ist es, wenn sich Pflegepersonen oder Ärzt:innen „zwischen den Fronten“ fühlen – z. B. zwischen den Erwartungen des Teams, der Patient:innen und deren An- und Zugehörigen.

Solche Situationen können zu einem Gefühl der Ohnmacht führen, insbesondere wenn unzureichende Ressourcen oder mangelnde Erfahrung des Personals hinzukommen. Zusätzlich kann die Angst, Patient:innen durch ehrliche Kommunikation zu entmutigen, dazu führen, dass ungewollt falsche Hoffnungen geweckt oder aufrechterhalten werden.

### Communication is key

Eine offen kommunizierte Orientierung kann hier Klarheit und Sicherheit schaffen. Transparenz gegenüber Patient:innen (sofern möglich) und An- und Zugehörigen ist essentiell. Die Änderung des Therapieziels sollte nicht als Abbruch der Behandlung verstanden werden, sondern als bewusste Anpassung an die neue Situation unter Beibehaltung einer hohen Versorgungsqualität und unterstützender Angebote wie Seelsorge oder anderer Berufsgruppen [8].

Bei Meinungsverschiedenheiten im Team oder mit An- und Zugehörigen kann wie oben beschrieben eine ethische Fallbesprechung initiiert werden. Eine regelmäßige Überprüfung der getroffenen Empfehlung ist ebenso vorgesehen wie die Möglichkeit der Ablehnung durch die Patient:innen und/oder ihr Umfeld mit dem Recht auf ärztlichen oder institutionellen Wechsel. Und wie so häufig im medizinischen Alltag, bleibt festzuhalten: Communication is key [9]!

### Ablauf der Klinischen Ethikberatung

Eine KEB folgt in der Regel einem strukturierten Ablauf. Nach Möglichkeit sollten Vertreter:innen aller beteiligten Berufsgruppen an der Beratung teilnehmen. Zu Beginn leitet die:der Moderator:in aus dem Team der KEB das Gespräch ein, erläutert kurz die Funktion und Rolle der KEB im konkreten Fallkontext, definiert den zeitlichen Rahmen und nennt den Anlass des Treffens.

Dann stellt die:der Fallinitiator:in die aktuelle Situation vor, gefolgt von einer strukturierten Sammlung der relevanten Informationen aus den unterschiedlichen Perspektiven der beteiligten Berufsgruppen – z. B. aus ärztlicher und pflegerischer Sicht, ergänzt durch die Sicht weiterer Berufsgruppen sowie, wenn möglich, aus Sicht der Patient:innen bzw. Vertreter:innen oder An- und Zugehörigen. Gegebenenfalls werden auch organisatorische, ökonomische und rechtliche Rahmenbedingungen einbezogen. Dann wird gemeinsam die wesentlichste ethische Frage formuliert.

Ziel der Moderation ist es, die möglichen Handlungsoptionen sichtbar zu machen und in einem ethischen Reflexionsrahmen zu analysieren. Dabei werden medizinethische Prinzipien wie Respekt vor der Autonomie der Patient:innen, Nichtschaden, Fürsorge und Gerechtigkeit zu den jeweiligen Optionen in Beziehung gesetzt.

Die Rolle der Moderation kann je nach Situation unterschiedlich ausgestaltet sein – sie reicht von einer rein reflektierenden bis zu einer stärker lösungsorientierten Haltung, von einer zurückhaltend vermittelnden bis zu einer proaktiv anwaltschaftlichen Position, z. B. zugunsten eines geäußerten Willens. Am Ende der Fallbesprechung, die aus praktischen Gründen zeitlich begrenzt sein ist, sollte zumindest eine gemeinsame Verständigung über das weitere Vorgehen stehen [10]. Dabei wird bewusst auf Begriffe wie „Lösung“ oder „Konsens“ verzichtet, da viele der besprochenen Fälle aufgrund ihres dilemmatischen Charakters keine einfache Lösung zulassen.

Stattdessen wird das Ergebnis der ethischen Beratung in Form einer Empfehlung beschrieben. Diese enthält nach Möglichkeit konkrete Aufgaben, Verantwortlichkeiten und ggf. Zeitvorgaben, nicht selten auch in Form eines Time Limited Trials (TLT).

### Time Limited Trial und Dokumentation

Ein TLT bezeichnet eine zeitlich begrenzte therapeutische Intervention, bei der eine medizinische Maßnahme mit klar definierten Zielen, Kriterien für Erfolg oder Misserfolg und einem festgelegten Beobachtungszeitraum durchgeführt wird.

Dieses Vorgehen bietet insbesondere in prognostisch unklaren Situationen die Möglichkeit, medizinische Maßnahmen unter kontrollierten Bedingungen zu erproben, ohne sich frühzeitig auf eine endgültige Therapieentscheidung festzulegen [11]. TLT fördert die Transparenz, ermöglicht eine schrittweise Entscheidungsfindung und dient sowohl dem Schutz der Patient:innenautonomie als auch der Orientierung des Behandlungsteams.

Die:der Moderator:in bzw. jemand aus dem Kreis der KEB erstellt im Anschluss zeitnah ein Protokoll des Beratungsgesprächs.

Dieses dokumentiert nicht nur das Ergebnis bzw. die Empfehlung, sondern auch den Anlass der Beratung, unterschiedliche Perspektiven, zugrunde liegende ethische und medizinische Argumentationslinien sowie relevante Abwägungen. Das Protokoll ist Teil der Patient:innenakte und kann allen Beteiligten – insbesondere den ärztlich Verantwortlichen – als Orientierung und Unterstützung, mitunter auch bei juristischen Auseinandersetzungen, dienen, da es ein Höchstmaß an Transparenz und Nachvollziehbarkeit schafft und insbesondere auch die breite Basis, auf der eine Therapieentscheidung fußt, belegt.

## Klinische Ethikkonsultationen: Ein- und Ausblicke aus den Vereinigten Staaten (Lorenz Faihs)

„I had a strong physical sensation of being in an aquarium: I could see out, the people outside could see me (if they chose to pay attention), but we were living and breathing in entirely different environments.“ (Hemon, 2011, S. 1)

Klinische Ethikberatungen entstanden in den 1970er‑Jahren in den Vereinigten Staaten als Reaktion auf neue ethische Herausforderungen, die durch medizinische Fortschritte wie Organtransplantation, Intensivpflege und lebenserhaltende Technologien hervorgebracht wurden [12]. Ein Blick in die Vereinigten Staaten kann aufgrund der weit verbreiteten Etablierung und der bestehenden Datenlage hilfreich sein, um einen besseren Einblick in die Funktion und Rolle der klinischen Ethikberatung zu erhalten und ihren Mehrwert für Patient:innen, ihre Angehörigen, das medizinische Personal und die Allgemeinheit darzulegen.

In den USA verlangen die Anforderungen der Joint Commission seit 1992 von allen akkreditierten Krankenhäusern einen Mechanismus zur Bearbeitung ethischer Fragestellungen im Bereich der medizinischen Versorgung [13].

Heutzutage sind klinische Ethikberatungen ein integraler Bestandteil des amerikanischen Gesundheitssystems und waren 2007 bereits in 81 % der US-amerikanischen Krankenhäuser und in allen Krankenhäusern mit über 100 Betten etabliert [14]. In den vergangenen zwei Jahrzehnten ist die Zahl der durchgeführten Ethikberatungen in den Vereinigten Staaten weiter um 94 % gestiegen. [15]. Derzeit werden jährlich rund 68.000 Konsultationen verzeichnet, was etwa 0,16 % aller stationären Aufnahmen entspricht, wobei die höchste Rate mit 0,88 % auf den Trauma-Intensivstationen zu beobachten ist [15, 16].

Die hohe Quote in diesem Fachbereich scheint aus der ethischen Komplexität der Fälle, der hohen Rate an entscheidungsunfähigen Patient:innen, den Fragestellungen bei Therapiezieländerungen und den Entscheidungen am Lebensende hervorzugehen [17–19]. Während auch in Österreich 87 % der Krankenhäuser angeben, ethische Konsultationen anzubieten, werden ihre Angebote jedoch noch nicht in demselben Ausmaß in Anspruch genommen [20].

Trotz kontextbedingter Unterschiede zeigen die ethischen Kernfragen im klinischen Alltag grundlegende Ähnlichkeiten. Diese Themen spiegeln die universellen Prinzipien der medizinischen Ethik wider, die der klinischen Praxis in verschiedenen Gesundheitssystemen zugrunde liegen: Respekt vor der Autonomie, Wohltätigkeit, Schadensvermeidung und Gerechtigkeit. Diese Prinzipien können in unterschiedlichsten Weisen und Situationen im Konflikt zueinander stehen und klinische Ethikberatungen werden aus verschiedensten Gründen zu klinischen Fällen hinzugezogen. In den Vereinigten Staaten betreffen Ethikkonsultationen häufig Situationen, in denen Unsicherheit über die Entscheidungsfähigkeit der Patient:innen besteht, Meinungsverschiedenheiten im Behandlungsteam auftreten oder Angehörige und Behandler:innen unterschiedliche Vorstellungen über das weitere Vorgehen haben [21–23].

Den meisten Anfragen liegt der Wunsch zur Konfliktlösung (34,6 %), zur Hilfe bei klinischen Entscheidungen oder Behandlungsplanung (13,1 %) oder zur Unterstützung in der Kommunikation der beteiligten Parteien (10 %) zu Grunde [21]. Zudem wird oftmals um Hilfestellung in emotional belastenden Situationen (8,9 %) ersucht [21]. Diese Daten legen nicht nur wiederkehrende ethische Konfliktkonstellationen dar, sondern verdeutlichen, dass die klinische Ethikberatung sich nicht nur auf außergewöhnliche Grenzfälle beschränkt, sondern als strukturierter Reflexionsraum dient, in dem ethische Spannungen im klinischen Alltag systematisch bearbeitet werden können.

Der Mehrwert von klinischen Ethikberatungen zeigt sich in verschiedenen Dimensionen. In empirischen Studien bewerteten zwischen 86–97 % der befragten Teilnehmenden die ethischen Konsultationen als hilfreich [5, 19, 24–27]. Bezüglich der Prozessqualität bewerteten über 86 % sowohl der Gesundheitsfachkräfte als auch der Angehörigen eine klinische Ethikberatung als nützlich bei der Identifikation ethischer Probleme, während etwa drei Viertel der Beteiligten über Fortschritte bei der Konfliktlösung berichteten [28, 29]. Besonders bedeutsam ist die Fähigkeit der klinischen Ethikberatung, Kommunikation zu fördern und Konsensbildung zu erleichtern, was in komplexen ethischen Behandlungssituationen von entscheidender Bedeutung ist. Klinisch manifestiert sich der Nutzen in einer reduzierten Verweildauer unheilbar kranker Patient:innen auf Intensivstationen [29–31]. Zudem zeigt sich eine hohe Umsetzungsrate von Beratungsempfehlungen zwischen 89,7–100 %, welche die praktische Relevanz und Akzeptanz der ethischen Beratung verdeutlicht [32].

Gleichzeitig dokumentieren die Studien eine Entlastung des Personals durch verringerte moralische Belastung bei 80 % der Pflegekräfte sowie ein gestärktes Selbstvertrauen der Beteiligten in ethisch herausfordernden Situationen [25, 33, 34]. Ökonomisch betrachtet konnten Ethikberatungen in einem beispielhaften US-amerikanischen Krankhaus, einer Studie zu Folge, Kosteneinsparungen von über $288.000 in sechs Monaten bewirken, mit einer geschätzten Kosteneinsparung von $1728 pro Patient:in durch klinische Ethikberatungen [35]. Wesentliche Kosteneinsparungen können durch die gezielte Vermeidung nicht-indizierter Therapien und verkürzte Krankenhausaufenthalte erzielt werden [29, 35].

Darüber hinaus fördern Ethikberatungen die ethische Kompetenz und Sensibilisierung des klinischen Personals, bieten einen geschützten Raum für moralische Reflexion und tragen zur Entwicklung und Implementierung von ethisch fundierten Leitlinien und Standards bei [29, 36, 37].

Klinische Ethikkonsultationen sind längst über den Status einer fakultativen Zusatzleistung hinausgewachsen und sind in Zeiten rasanten wissenschaftlichen und technischen Wandels ein unverzichtbarer Bestandteil der modernen Gesundheitsversorgung. Sie sind Ausdruck einer Kultur, die die Bedeutung ethischer Reflexion in der Medizin ernst nimmt und strukturell verankert. Die Erfahrungen aus den Vereinigten Staaten zeigen, dass Ethikberatungen einen messbaren Nutzen für Patient:innen, Angehörige, Gesundheitspersonal und Gesundheitssysteme haben. Für die Zukunft wird es entscheidend sein, diese Angebote in unterschiedlichen Kontexten weiter zu adaptieren, methodisch fundiert zu evaluieren und Strukturen zu etablieren, die eine dauerhafte Verankerung und Weiterentwicklung klinischer Ethik im klinischen Alltag ermöglichen.

Die klinische Ethik befasst sich mit individuellen Schicksalen und Ethikberatungen dienen dabei als wichtiges Instrument, das im Rahmen des ethischen Ökosystems einen zentralen Beitrag zur Gestaltung und Wahrung des moralischen Klimas im Gesundheitswesen leisten kann.

## Mittendrin und außen vor? Perspektiven der Pflege auf ethische Herausforderungen im Klinikalltag (Sabine Pleschberger, Jacob Roth)

In Österreich sind Berufsangehörige der Gesundheits- und Krankenpflege die größte Berufsgruppe in Krankenhäusern [38]. Dadurch sind sie nicht nur allgegenwärtig im Versorgungsprozess, sondern auch maßgeblich an der Schnittstelle zwischen Patient:innen, Ärzt:innen, Therapeut:innen und Angehörigen tätig. Kein anderer Beruf arbeitet so nah und so viel direkt an bzw. mit den Menschen wie die professionelle Pflege. Pflegende sind also „mittendrin“ in der Versorgung, und dennoch erleben sie sich oft als „außen vor“, wenn es um entscheidende ethische Fragestellungen geht.

Dass Pflegefachpersonen trotz ihrer zentralen Rolle häufig nicht in ethische Entscheidungsprozesse eingebunden werden, ist kein neues Phänomen und auch kein spezifisch österreichisches: Studien zeigen, dass sie sich insbesondere bei Fragen zu Therapiezielen und der Behandlung am Lebensende häufig übergangen fühlen [39, 40]. Die Ursachen dafür sind vielschichtig, und können auch in der Professionsgeschichte aufgespürt werden: eine im Vergleich zu manchen Ländern verspätete Akademisierung der Ausbildung, die noch junge Geschichte der Disziplin der Pflegewissenschaft, die Trennung zwischen Medizin und Pflege, ein unscharfes Berufsbild und das historische Erbe eines als weiblich konnotierten „Helferinnenberufes“ gehören dazu [41, 42]. Zudem werden viele Tätigkeiten in Pflegeberufen gar nicht als Pflege im engeren Sinne bezeichnet und die Arbeit häufig als nachgeordnet oder medizinisch fremdbestimmt wahrgenommen [42]. Obwohl sich die gesellschaftliche Wahrnehmung der beruflichen Pflege wandelt, bleibt das professionelle Selbstverständnis somit im Spannungsfeld zwischen traditionellen Rollenzuschreibungen und modernen Kompetenzanforderungen verhaftet.

### Pflege als ethisch reflektierende und reflektierte Gesundheitsprofession

Das professionelle Selbstverständnis der beruflichen Pflege orientiert sich am Zentralwert „Gesundheit“ [43]. Dieser prägt sowohl die praktische als auch die ethische Dimension des Berufs. Das aktuelle Gesundheits- und Krankenpflegegesetz (GuKG) sowie internationale Standards, wie der Ethik-Kodex des International Council of Nurses (ICN), sind wichtige Bezugsrahmen für ein professionelles Berufsverständnis und verankern die ethische Dimension fest in der Professionsidentität [44, 45].

Das Kompetenzmodell für Gesundheits- und Krankenpflegeberufe der Gesundheit Österreich GmbH, das die Grundlage für das GuKG bildet und sich vom standesethischen Bezugsrahmen ableitet, gliedert sich in drei wesentliche Säulen: die Grundhaltungen der professionellen Pflege, die Bereitstellung von Pflege sowie die Prozessgestaltung und die Entwicklung und Sicherung von Qualität [46]. Eine besonders hervorzuhebende Grundhaltung darin ist das ethische Handeln. Es verpflichtet Pflegefachpersonen, sich kontinuierlich mit den ethischen Fragestellungen ihres beruflichen Handelns auseinanderzusetzen. Diese Auseinandersetzung fördert eine reflektierte Praxis und macht das an ethische Reflexion gebundene pflegerische Handeln zu einem wertvollen Bestandteil der interdisziplinären Zusammenarbeit im klinischen Versorgungsprozess. Pflegefachpersonen sind in die Umsetzung medizinischer und pflegerischer Interventionen im Versorgungsprozess eingebunden. Insbesondere im akutstationären Bereich sind ihre Aufgaben eng mit medizinisch-diagnostischen sowie medizinisch-therapeutischen Maßnahmen und Tätigkeiten zur Behandlung verbunden (§§ 15, 15a, 83, 83a GuKG). Allerdings geraten durch die Vordergründigkeit der medizinnahen Tätigkeiten die eigentlichen pflegerischen Kernkompetenzen (§ 14 GuKG) zunehmend unter Druck [47, 48].

Dabei bietet die Schaffung pflegeprofessionseigener Entfaltungsräume, die sich durch Proximität – also die bewusst gestaltete physische, relationale und emotionale Nähe – sowie Interaktionsorientierung gegenüber den Patient:innen auszeichnen, ein beträchtliches Potenzial für eine qualitätsvolle Auseinandersetzung mit ethisch herausfordernden Situationen [49]. Denn durch die Nähe und Interaktion mit den Patient:innen können deren relevante Bedürfnisse und Wünsche umfassend wahrgenommen und berücksichtigt werden. Dies erscheint für eine gute Versorgungsqualität und sinnvolle Therapiezielsetzung von zentraler Bedeutung [50].

Pflegefachpersonen sind häufig die ersten, die ethische Spannungen und Konflikte wahrnehmen. Ihre Nähe zu den Patient:innen und ihre detaillierten Einblicke in deren Zustand und Wünsche prädestiniert Pflegefachpersonen als Advokat:innen für Patient:innen – vor allem für diejenigen, die nicht in der Lage sind, sich selbst zu artikulieren. Advocacy ist ein zentraler ethischer Grundsatz der Pflege und im Ethikkodex des ICN verankert. Dort heißt es: „Die Pflegefachperson als Fürsprecher:in unterstützt aktiv eine richtige und gute Sache; unterstützt andere darin, sich für sich selbst oder für andere einzusetzen, die nicht für sich selbst sprechen können. Die Fürsprache erfolgt immer mit Zustimmung der betroffenen Person.“ [45, S. 24].

### Rolle der Pflege in Ethikberatung – rechtlich verankert, faktisch ausbaufähig

In Österreich hat der Gesetzgeber die konzeptionell wichtige Rolle der Pflege anerkannt: In § 16 GuKG wird die Mitwirkung an ethischen Entscheidungsprozessen ausdrücklich erwähnt. Pflegende sind damit nicht nur berechtigt, sondern auch verpflichtet, sich aktiv einzubringen. Ihre Beteiligung umfasst konzeptionell verschiedene zentrale Aufgaben: Die professionelle Pflege erkennt a) den Bedarf an einer ethischen Beratung (EB), beruft diese ein und trägt dafür Sorge, dass zeitnah ein entsprechender Prozess eingeleitet wird. Zudem kann sie b) die Organisation und Koordination der EB übernehmen. Als Fachpersonen c) bringen sie sich mit umfassendem Wissen über die jeweilige Situation und den Fall in den Entscheidungsprozess ein. Schließlich nimmt sie als Vertreter:in der Berufsgruppe d) aktiv an der EB teil und bringt ihre Perspektive als Teil eines interprofessionellen Teams mit. Dabei bringt sie insbesondere den Blick auf die Versorgung und den ethischen Bezugsrahmen ein [51].

Es gibt Hinweise darauf, dass wissenschaftlich dokumentierte Probleme – wie die sprachliche und strukturelle Prägung der Ethikberatung durch das bio-medizinische Paradigma – weiterhin bestehen. Diese Prägung führt dazu, dass Beiträge der Pflege entweder entwertet oder gar nicht berücksichtigt werden, wodurch das Potenzial einer aktiven Einbindung der Pflege oftmals ungenutzt bleibt [52]. Womit wertvolle Perspektiven verloren gehen – zum Nachteil einer umfassenden, interprofessionellen Ethikberatung.

### Voraussetzungen für eine gelingende Einbindung

Damit die Pflege ihre Rolle in Ethikprozessen wirkungsvoll ausfüllen kann, bedarf es an Maßnahmen auf unterschiedlicher Ebene: Zielgerichtete Aus- und Weiterbildung von Ethikkompetenzen, Möglichkeit und Bereitschaft zur aktiven Teilnahme an Arbeitsgruppen, Ethikberatungen, Intervisionen oder ethischen Fallreflexionen. Diese Maßnahmen müssen auf struktureller Ebene der Organisation und des Systems, auf professioneller Ebene der Berufsgruppe sowie auf individueller Ebene geschaffen und gefördert werden [53–55]. Ethikberatung ist somit auch ein wichtiges Instrument zur Qualitätssicherung und zur Prävention von moralischem Stress, dem viele Pflegende durch ihre erlebte Ohnmacht im System ausgesetzt sind [56].

### Conclusio

Angesichts der vielen Herausforderungen – demografischer Wandel, Personalknappheit, technologische Entwicklungen – wird Ethikberatung weiter an Bedeutung gewinnen. Ein effektiver Umgang mit der damit verbundenen Unsicherheit und Komplexität erfordert eine strukturierte und interdisziplinäre Herangehensweise. Die vielleicht wichtigste Ressource von Ethikgremien ist demnach wohl die Zusammensetzung aus Mitgliedern unterschiedlicher Berufsgruppen [51]. Pflegefachpersonen sind aufgrund ihrer pflegerischen Fachkompetenz, ihrer ethischen Reflexionsfähigkeit, ihrer Advocacy-Praxis, ihrer Proximität und ihrer Interaktionsorientierung unverzichtbare Partner:innen in interdisziplinären Ethikberatungen. Es reicht nicht aus, die professionelle Pflege nur als Teil der Umsetzung zu begreifen, sondern sie muss als essenzielle Mitgestalterin ethischer Prozesse begriffen werden. Denn sie ist – berufsethisch wie praktisch – mittendrin.

## Indikationsstellung und Therapiezieländerung – Herausforderungen für Chirurgie und Intensivmedizin (Bruno K. Podesser, Helga Dier)

Zunehmend ältere und kränkere Patient:innen stellen eine immer größere Herausforderung für Chirurgie, Anästhesie und Intensivmedizin dar. In diesem Artikel versuchen wir die Bedeutung der Ethik in diesem Spannungsfeld in den Vordergrund zu rücken. Damit werden medizinische Kernbegriffe wie Therapieziel, Indikation und Entscheidungsprozesse mit patientenspezifischen Eigenschaften und Zielen verbunden. Dies stellt Patient:innen, Angehörige und die Gesundheitsberufe vor schwierige Entscheidungen. Hier können klinisch-ethische Beratungsstellen eine wichtige Hilfe leisten.

### Einleitung

In Folge der zunehmend älteren Patient:innenpopulation mit immer mehr Ko-Morbiditäten gerät die Chirurgie und die mit ihr eng verbundene Anästhesie und Intensivmedizin vermehrt unter Druck. Trotz immer schonenderer und „minimal-invasiverer“ Verfahren können nicht alle Therapieziele erreicht werden und müssen diese oft mit den Patient:innen oder Angehörigen diskutiert und womöglich adaptiert werden. Treten Entscheidungen an die Stelle technischer Abläufe, rückt die Ethik in den Vordergrund: Welche Ziele verfolgt der Eingriff? Welche Indikation rechtfertigt ihn? Welche Werte sollen die Entscheidung tragen, wer muss beteiligt werden, und wie wird Transparenz gewährleistet? Ethik dient hier nicht als Bremse, sondern als Orientierungssystem, das Patient:innensicherheit, Würde und Gerechtigkeit in den Mittelpunkt stellt.

Die Kernbegriffe Therapieziel, Indikation und Entscheidungsprozesse verzahnen sich zu einem Entscheidungsrahmen, der sowohl medizinische Evidenz als auch individuelle Lebensentwürfe berücksichtigt [57].

### Methoden und Ergebnisse

Im Folgenden sollen Begriffe wie Therapieziel und Indikation definiert werden. Danach werden praxis-relevante Checklisten für Ärztinnen und Ärzte angeboten [58].

### Therapieziel: Was soll erreicht werden?

Definition: Das Therapieziel beschreibt den Endzustand nach einer Intervention und berücksichtigt gesundheitliche, funktionale, psychosoziale und existentielle Dimensionen. Es umfasst Heilung, Symptomlinderung, Funktionsverbesserung, Lebensqualität sowie rein-palliative Ziele (Schmerz- und Belastungsreduktion, Stabilisierung der Lebensqualität; keine Heilungsversprechen).

#### Zielkonsistenz

- Realismus: Ziele müssen erreichbar sein, basierend auf Evidenz, Vorerkrankungen und individuellen Gegebenheiten.
- Patientenzentrierung: Werte, Bedürfnisse und Präferenzen der/des Patient:in berücksichtigen.
- Messbarkeit: Objektive Zielgrößen (z. B. Schmerzskala, Funktions-Scores, Alltagsfähigkeit).

#### Zieldefinition

- Übergeordnetes Ziel: Nutzen der Intervention.
- Zwischenziele: Schrittweise Etappen (z. B. Schmerzreduktion in 4–6 Wochen; Beweglichkeit in 3 Monaten).
- Minimal‑/Maximalziel: Transparenz über Unsicherheiten.
- Erfolgskriterien: Konkrete Messgrößen (z. B. Schmerz Numerische Rating Skala (NRS) ≤ 2/10, Funktions-Score, Rückkehr zur Alltagsaktivität).

#### Zielarten

- Heilungs‑/Funktionsziele: Heilung, Symptomlinderung, Funktionsverbesserung.
- Lebensqualitätsziele: Reduktion der Belastung, bessere Alltagsbewältigung, psychische Entlastung.
- Rekonstruktiv/ästhetische Ziele: Funktionale und ästhetische Restoration.
- Palliative Ziele: Schmerz- und Belastungsreduktion, Stabilisierung der Lebensqualität; keine Heilungsversprechen.

#### Timing, Lebenswelt und Ressourcen

- Lebensumstände: Beruf, Familie, soziales Umfeld.
- Psychische Verfassung: Motivation, Erwartungshaltungen, Rehabilitationsbereitschaft.
- Ressourcen: Verfügbarkeit von Therapien, Rehabilitations-Maßnahmen, Pflegeunterstützung.

#### Dynamik der Zielsetzung

- Iteration: Ziele bei neuen Informationen/Umständen anpassen.
- Risiko-Nutzen-Balance: Zielmodifikation bei Befunden, um Überforderung zu vermeiden.
- Kommunikation: Änderungen transparent kommunizieren und gemeinsam entscheiden.

#### Grenzen des Therapieziels

- Vermeidung unerreichbarer/überzogener Ziele.
- Würde, Autonomie und Ethik wahren; Grundwerte nicht kompromittieren.
- Abgleich mit Optionen, inkl. nicht-operativen Ansätzen und Alternativen.

#### Checkliste für Ärzt:innen vor einem Eingriff

- Ist ein klares Ziel formuliert und mit der/dem Patient:in abgestimmt?
- Sind Zwischenziele, Minimal‑/Maximalziel und Erfolgskriterien definiert?
- Welche Zielarten werden verfolgt (heilend, funktionsbezogen, lebensqualitätsorientiert, palliativ)?
- Passt das Ziel realistisch zu Evidenz, Vorerkrankungen und Lebensumständen?
- Wurden Ressourcen (Rehabilitation, Pflege, Unterstützung) eingeplant?
- Gibt es einen Plan für Zielanpassungen bei neuen Befunden oder Komplikationen?
- Wurde eine klare Kommunikation über Behandlungsgrenzen und Alternativen geführt?
- Will die Patient:in dieses Therapieziel erreichen?

Wenn das Therapieziel nicht erreicht werden kann und nach alternativen Zielen gesucht wird, muss die Indikation nochmals diskutiert werden.

### Indikation: Warum ist der Eingriff sinnvoll?

Definition: Eine medizinische Indikation stellt eine fachlich begründete Einschätzung dar, dass eine Therapiemaßnahme geeignet und nützlich ist, um ein bestimmtes (realistisches) Therapieziel mit einer gewissen Wahrscheinlichkeit zu erreichen. Indikationen basieren auf klinischen Befunden, Diagnostik, Prognose und dem zu erwartenden Benefit-Risiko-Verhältnis.

#### Kategorien der Indikation

- Absolute Indikation: Medizinisch zwingend, z. B. akute lebensrettende Eingriffe.
- Relative Indikation: Nutzen überwiegt Risiko, Patient:in stimmt zu (z. B. funktionelle Verbesserungen, Lebensqualität).
- Grenzindikationen: Unsicherheit über Prognose oder Nutzen, sorgfältige Abwägung erforderlich.

#### Checkliste für Ärzt:innen mit entscheidungsrelevanten Faktoren

- Evidenzbasis: Stärke der wissenschaftlichen Grundlage.
- Patientenvoraussetzungen: Alter, Begleiterkrankungen, Operationsrisiken, Rehabilitationsmöglichkeiten.
- Alternative Szenarien: Könnte eine weniger invasive oder konservative Behandlung genauso wirken?
- Timing: Passt der Zeitpunkt in Lebensumstände, Psychologie und soziales Umfeld?

Steht nach reiflicher Überlegung die ärztliche Indikation, so ist dies eine individuelle und streng fallbezogene Entscheidung, bei der die Nutzen-Schadensabwägung im Vordergrund ist und der Nutzen klar über dem Schaden steht.

### Entscheidungsprozesse in der Praxis

#### Autonomie und informierte Einwilligung

- Information: Patient:innen müssen verständlich über Ziel, Nutzen, Risiken, Alternativen und Unsicherheiten aufgeklärt werden.
- Freie Entscheidung: Keine Druckausübung; Raum für Bedenkzeit und Folgegespräche.
- Verifikation des Verständnisses: Rückfragen, Wiederholung der Kernaussagen, ggf. schriftliche Zusammenfassungen.

#### Benefizienz, Non-Malefizienz und Verhältnismäßigkeit

- Benefizienz: Eingriff soll der/dem Patient:in nutzen.
- Non-Malefizienz: Risiken minimieren, unnötige Schäden vermeiden.
- Verhältnismäßigkeit: Nutzen-Risiko-Abwägung muss sinnvoll und fair sein, besonders bei großen Operationen.

#### Nicht-entscheidungsfähige Patient:innen – Welche Fragen müssen gestellt werden

- Liegt ein antizipierter Patient:innenwille vor (Patientenverfügung)?
- Liegen klare Vertretungsinstrumente wie zum Beispiel eine Vorsorgevollmacht vor?
- Was ist der mutmaßliche Patient:innenwille?
- „In dubio pro vita“ – also im Zweifelsfall immer für das Leben.
- Substituted judgment standard – also eine Entscheidung im Sinne des/der Betroffenen.

#### Entscheidungen treffen

- Entscheiden können heißt entscheiden müssen.
- Entschieden haben heißt verantworten müssen.
- Verantworten müssen heißt begründen können.
- Daher die Fragen:
- Wurde auf die Sichtweise aller Betroffenen ausreichend eingegangen?
- Welche Optionen sind im Konkreten am ehesten gerecht?
- Wie lautet das stärkste Gegenargument?
- Kann es entkräftet werden?

### Diskussion

Spätestens im Rahmen von komplexen Situationen innerhalb eines Prozesses, bei dem man versucht, entsprechend bekannter Wertvorstellungen und geäußerter Willensbekundungen eines/einer Patient:in eine Entscheidung im Sinne der Betroffenen zu finden (substituted judgment standard [59]) sowie bei widersprüchlichen Wahrnehmungen in Bezug auf medizinisch indizierte oder nicht-indizierte Maßnahmen, ist eine aktive Involvierung der klinischen Ethikberatung anzustreben.

In Situationen mit begrenzten Ressourcen oder hochriskanten Therapien rücken Prinzipien wie Verhältnismäßigkeit, Gerechtigkeit und Würde stärker in den Vordergrund. Klinisch-ethische Beratungsstellen und strukturierte Fallbesprechungen unterstützen den Entscheidungsprozess. Dies kann nur in interdisziplinärer Kooperation zwischen Chirurgie, Anästhesiologie, Intensivmedizin, Palliativmedizin sowie Pflege- und Sozialdienst gelingen. Gemeinsam mit den Angehörigen müssen Zielrahmen, Indikation und Ressourcen bedacht werden.

Nicht-operative Optionen sollten frühzeitig diskutiert werden, um unnötige invasive Maßnahmen zu vermeiden. Dabei gilt, dass die Definition von Erfolgskriterien patientenzentriert sein sollte (z. B. Rückkehr zur Alltagsaktivität, Schmerzreduktion, Unabhängigkeit) [60]. Eine frühzeitige Ressourcenplanung wie Rehabilitationsmöglichkeiten, häusliche Pflege, soziale Unterstützung und Finanzierungsaspekte beeinflussen die Realisierbarkeit von Therapiezielen.

## Vom kurativen zum palliativen Setting: Ein Fallbericht zur multiprofessionellen Entscheidungsfindung mit Unterstützung durch die Klinische Ethikberatung bei einem Patienten mit supraglottischem Larynxkarzinom (Joachim Widder)

Ein 82-jähriger Patient mit einem supraglottischen Larynxkarzinom (cT2 cN2c M0) wurde nach Abschluss einer kurativ intendierten Radiotherapie in ein palliatives Betreuungskonzept übergeleitet, obwohl der Tumor möglicherweise unter Kontrolle war. Der Verlauf verdeutlicht die Herausforderungen bei der interdisziplinären Entscheidungsfindung am Lebensende, insbesondere bei kognitiver Fluktuation, divergierenden Patienten- und Angehörigenwünschen sowie ethisch sensiblen Fragestellungen zur künstlichen Ernährung. Der Patient verstarb letztlich zu Hause, zehn Wochen nach der Entlassung.

### Anamnese

Der Patient, 82 Jahre alt, stellte sich mit einer anhaltenden Schluckstörung als Folge eines supraglottischen Larynxkarzinoms vor. Eine kurativ intendierte Radiotherapie über sieben Wochen wurde abgeschlossen. Weitere relevante Diagnosen umfassten eine chronisch obstruktive Lungenerkrankung (COPD), eine Chemotherapie-induzierte Polyneuropathie sowie ein mehrere Jahre zurückliegendes nicht-kleinzelliges Lungenkarzinom (NSCLC) ohne Hinweis auf aktuelles Rezidiv.

Nach Abschluss der Radiotherapie zeigte sich ein erwartbarer, aber signifikanter Abfall des Allgemeinzustandes. Der Patient war prinzipiell mobil, wies jedoch ein relevantes Selbstfürsorgedefizit sowie eine deutliche Dysphagie mit wiederholten Aspirationspneumonien auf. Eine ausreichende orale Ernährung war wegen einer Dysfunktion des Schluckapparats nicht mehr möglich. Eine parenterale Ernährung wurde intermittierend durchgeführt.

### Prognose

Das Wiedererlangen der Schluckfähigkeit wurde als sehr unwahrscheinlich eingeschätzt. Die Tumorprognose war zum damaligen Zeitpunkt noch offen, da eine verlässliche Beurteilung erst einige Zeit nach Abschluss der Radiotherapie möglich ist.

Ein operatives Vorgehen im Sinne einer Laryngektomie war vom Patienten bereits vor Beginn der Therapie ausdrücklich abgelehnt worden.

### Klinische Ethikberatung und Entscheidungsfindung

Indikation für die Klinische Ethikberatung war die Diskrepanz zwischen dem geäußerten Wunsch des Patienten nach Entlassung in die Häuslichkeit trotz Schluckdysfunktion und daraus resultierend insuffizienter Ernährung, dem Wunsch der Ehefrau nach Weiterbehandlung im stationären Setting und der Einschätzung des Behandlungsteams, dass eine perkutane endoskopische Gastrostomie (PEG) zur Ernährung medizinisch indiziert wäre.

Drei psychiatrische Konsile zeigten zuletzt Hinweise auf ein hyperaktives Delir mit multifaktorieller Genese. Die Einsichtsfähigkeit in die Erkrankung war eingeschränkt, jedoch bestand keine akute Eigen- oder Fremdgefährdung. Suizidalität wurde verneint.

Der Patient lehnte alle vorgeschlagenen ernährungsmedizinischen Maßnahmen (nasogastrale Sonde, PEG, logopädisches Schlucktraining) sowie weitere diesbezügliche Untersuchungen ab, da er von einer Spontanbesserung ausging.

Die Klinische Ethikberatung empfahl den Wechsel vom kurativen in ein palliatives Behandlungskonzept unter Beachtung des Patientenwillens, auch wenn die Tumorprognose – eine komplette Remission war durchaus noch möglich – noch nicht deutlich war. Die Organisation einer Entlassung nach Hause sowie eine Betreuung durch ein mobiles Palliativteam wurde angeregt. Zuvor sollten der Patient und seine Ehefrau umfassend über die Situation, das wahrscheinlich nahende Lebensende und die verfügbaren Unterstützungsangebote aufgeklärt und aktiv in die Planung einbezogen werden. Die Inhalte des Gespräches wurden im Rahmen eines Ethik-Protokolls dokumentiert und sind Teil der elektronischen Patientenakte.

### Nachsorge und Verlauf

Zehn Wochen nach Entlassung erfolgte eine ambulante Nachsorgeuntersuchung an der Abteilung für Hals‑, Nasen- und Ohrenkrankheiten und der radioonkologischen Abteilung. Der Patient erschien im Sitzwagen, begleitet von seiner Ehefrau. Trotz reduzierten Allgemeinzustandes, Gewichtsverlusts (von 75 auf 63 kg) und chronischer Fatigue äußerte er weiterhin keine Bereitschaft zur Nutzung von hochkalorischer Zusatznahrung oder zur parenteralen Ernährung.

Subjektiv war er beschwerdefrei im Bereich des Larynx. Die Tumornachsorge zeigte keinen Hinweis auf Tumorresiduum, also eine komplette Remission. Die Ehefrau übernahm die Pflege zu Hause und bereitete Breikost und Suppen zu, die jedoch nur in kleinen Mengen aufgenommen wurden.

Diagnostisch zeigten sich in der Bildgebung ein Pleuraerguss sowie Verdichtungen im linken Unterlappen, differenzialdiagnostisch als durch Aspiration bedingt interpretiert. Die Ehefrau wünschte die Fortführung regelmäßiger computertomographischer Kontrollen. Eine weitere Computertomographie wurde für drei Monate später geplant.

Ein Tag nach dem ambulanten Termin informierte die Ehefrau das Krankenhaus telefonisch über den Tod des Patienten in der häuslichen Umgebung.

### Diskussion

Dieses Patientenbeispiel illustriert die Herausforderungen in der klinischen Praxis, wenn Patientenwille, Angehörigenmeinung und medizinische Indikation divergieren. Besonders relevant ist der Wechsel von kurativem zu palliativem Setting, welcher nicht nur medizinisch, sondern auch ethisch, kommunikativ und organisatorisch gut begleitet sein muss und in diesem Fall eben nicht durch eine unkontrollierte Tumorerkrankung bedingt war, sondern durch Folgeerscheinungen der Tumorremission (Zerstörung des Kehldeckels) und durch Nebenwirkungen der Radiotherapie (Störung des Schluckapparats) bei Weigerung des Patienten, andere Ernährungswege zuzulassen.

Die konsequente Berücksichtigung des Patientenwillens steht im Zentrum der Entscheidungsfindung, auch wenn dieser mit erheblichen Risiken – wie Mangelernährung und Aspiration – einhergeht. Wichtig ist hierbei die fortlaufende Information und Begleitung der Angehörigen, um die Tragweite der Entscheidungen verständlich zu machen.

Die Betreuung durch ein multiprofessionelles Team sowie ein mobiles Palliativteam ermöglichte trotz schwieriger Rahmenbedingungen eine Betreuung im häuslichen Umfeld und letztlich ein Sterben in der gewählten Umgebung.

### Fazit für die Praxis

- Der Patientenwille ist bei entsprechender Urteilsfähigkeit vorrangig, auch wenn daraus resultierende Risiken bestehen.
- Eine Klinische Ethikberatung kann angesichts einer komplexen Situation helfen, Behandlungskonflikte zu moderieren.
- Palliative Betreuung soll individuell, multiprofessionell und lebensnah erfolgen.
- Angehörige benötigen klare Information und emotionale Begleitung, insbesondere im häuslichen Setting.
- Die Frage, „Wie viel Ernährung ist am Lebensende notwendig?“, bleibt ethisch herausfordernd.

## Wozu wir alle Ethik in der Medizin brauchen (Barbara Friesenecker)

Die rasante technologische Entwicklung hat die Medizin in eine Ära nahezu unbegrenzter Machbarkeit geführt. Wenn früher Sterben als unausweichlich galt, ist heute auch für schwer erkrankte Patient:innen die Rückkehr in ein qualitätvolles Leben möglich. Allerdings führt eine rein an der Machbarkeit orientierte Medizin zunehmend zum Verlust von Menschlichkeit – *Übertherapie* ist zunehmend Tür und Tor geöffnet [61–63]. Neben der rein klinisch-wissenschaftlichen Beurteilung der Patient:innen ist daher für das Treffen schwieriger therapeutischer Entscheidungen eine fundierte ethische Reflexion mit dem Ziel, Entscheidungsprozesse patientenorientiert, gerecht [64] und verantwortungsvoll zu gestalten, eine unabdingbare ärztliche Verpflichtung.

Die Indikation für eine Behandlung darf sich keinesfalls rein über die technische Machbarkeit definieren, sondern muss sich immer an einem individuellen und realistischen Therapieziel orientieren [65]. Ist *Heilung* nicht mehr erreichbar, muss rechtzeitig eine *Therapiezieländerung* (TZÄ) Richtung Palliation getroffen werden [66]. Die Fortführung einer medizinischen Handlung ohne Indikation oder gar gegen den wahren oder mutmaßlichen Patien:innenwillen wäre eine Körperverletzung und damit eine strafbare Handlung.

Patient:innen haben das Recht, Behandlungen ohne Angabe von Gründen abzulehnen, selbst wenn diese medizinisch indiziert wären (Recht auf Unvernunft). Ärzt:in hat die Pflicht, Patient:in nachweislich (schriftlich; mit Zeug:in) über die negativen Konsequenzen bis hin zum Tod zu informieren [67].

### Ethische Prinzipien und Entscheidungsmodelle

Eine strukturierte ethische Reflexion kann über das 2‑Säulen-Modell geführt werden: 2 gleichberechtigte Säulen – Säule der Autonomie (Patient:in) und Säule der Indikation (Ärzt:in) – regeln, ob eine Behandlung stattfindet oder nicht [68]. Die *Prinzipienethik* nach Beauchamp und Childress definiert vier medizinethische Prinzipen (Autonomie, Wohltun, Nicht-Schaden und Gerechtigkeit), die gleichwertig nebeneinanderstehen und in Konflikt geraten können – die Balance der Prinzipien ist abzuwägen [69]. Autonomie findet nicht immer angemessene Berücksichtigung, insbesondere wenn keine schriftliche Verfügung vorliegt. Kommunikationsdefizite bei Aufklärungsgesprächen verhindern einen korrekten *informed consent*. Kenntnisse in Medizinethik und -recht (z. B. Ärztegesetz, Erwachsenenschutzgesetz, Patientenverfügungsgesetz [67, 70, 71]) sowie die Fähigkeit zu einer mitfühlenden, aber unmissverständlichen Kommunikation sind daher Voraussetzung für eine gute interdisziplinäre und multiprofessionelle Zusammenarbeit.

Sie schaffen die Basis für die Erarbeitung einer Lösung im besten Sinne der Patient:in (*shared decision making) *[72, 73]. Bereits im Studium die Grundlage für ein medizinethisches, -rechtliches Bewusstsein zu legen, kann die Angst vor medicolegalen Konsequenzen reduzieren. An der Medizinischen Universität Innsbruck wird im Pflichtcurriculum Humanmedizin in allen 3 Studienabschnitten praxisbezogen Ethik gelehrt [74]. Zusammen mit einer verpflichtenden postpromotionellen Weiterbildung in Ethik, Recht, Palliativmedizin und Kommunikation könnte dies zukünftig zur Vermeidung von Übertherapie beitragen, um damit die negativen Folgen für Patient:innen (Chronisch kritische Erkrankung) [75] und Angehörige (Posttraumatisches Stresssyndrom) zu reduzieren [76], das *Burnout Risiko* bei medizinischem Fachpersonal zu verringern [77–79] und die *Kostenexplosion* im Gesundheitssystem einzubremsen.

### Patientenbeispiel

Nach einer langjährigen Krankengeschichte ist der 82 Jahre alte Patient (P) erschöpft, gebrechlich, meist bettlägerig und lehnt bei stark reduziertem Allgemein- und Ernährungszustand jegliche weiteren belastenden oder invasiven Therapien/Diagnostik ab. Sein Wunsch ist Entlassung nach Hause, um dort seine letzte Lebensphase im Beisein seiner Frau zu verbringen. Dies kann er klar und wiederholt äußern, obwohl er psychiatrisch *fraglich entscheidungsfähig *ist – autopsychisch ist P vollständig orientiert und nicht suizidal.

P sagt klar, was er NICHT will: Sondenernährung oder kalorisch aufgewertete Kost, Tracheostoma, Abklärung einer bestehenden Schluckstörung, Logopädie, Rehabilitationsmaßnahmen und Raucher-Entwöhnung. Obwohl er aufgrund von Dysphagie wegen Aspirationspneumonien mehrfach stationär aufgenommen war, bedeutet Lebensqualität in dieser letzten Lebensphase für ihn, zu Hause zu sein und selber das zu essen, was seine Frau für ihn kocht – so gut es eben geht.

Die Ehefrau hat durch die jahrelangen Erkrankungen ihres Mannes viel mitgemacht. Sie hat Angst, P alleine zu Hause zu betreuen; über das Sterben hat man mit ihr nicht explizit gesprochen. Sie wünscht weitere stationäre Betreuung, Abklärung und Behandlung, wahrscheinlich auch, weil das für sie selbst ein *„sicheres Szenario“* ist.

#### Das Behandlungsteam

Prognostisch ist die Wiedererlangung eines suffizienten Schluckaktes nicht zu erwarten, eine kurative Therapieoption besteht nicht. P ist zur Zeit tumorfrei, weswegen es dem medizinischen Team schwerfällt, auf die Wünsche von P offen einzugehen, sein wahrscheinlich baldiges Sterben anzuerkennen und ihn diesen Weg auf seine Weise gehen zu lassen!

Der weitgehende Verzicht auf Nahrung und Flüssigkeit macht aus medizinischer Sicht ein „besser werden“ und „weiterleben“ unmöglich, trotzdem werden P wiederholt auf Besserung ausgerichtete Therapievorschläge unterbreitet.

Der Wunsch des als „ansprechbar“ und „kommunikationsfähig“ beschriebenen P, seine letzte Zeit bis zum Sterben selbst gestalten zu wollen, wird vom behandelnden Team nicht ausreichend wahrgenommen. Sein nie klar geäußerter, aber im Wünschen und Handeln versteckter Sterbewunsch wird verkannt und nicht thematisiert – weswegen darüber auch mit seiner Frau nicht offen gesprochen wird. Das Team fühlt sich von P zurückgewiesen. Klinisches Handeln wird bestimmt von der Sorge vor dem Vorwurf der Unterlassung.

Die Diskrepanz zwischen dem Willen von P (Entlassung nach Hause, sterben), der Ehefrau (weitere stationäre Behandlung, „Sicherheit“) und des Behandlungs-Teams (kurative Vorwärtsstrategie) führt zur Involvierung der Klinischen Ethikberatung (KEB), die einen Wechsel von kurativem zu palliativem Setting vorschlägt. Nach einem intensiven Gespräch, im Rahmen dessen auch die Folgen der vom P gewünschten oralen Nahrungsaufnahme (hypokalorische Ernährung, Aspirationspneumonie) angesprochen werden, wird die Entlassung nach Hause geplant. P und seine Ehefrau erhalten Unterstützung durch ein mobiles Palliativteam. Als P nach 10 Wochen zu einer Routine-Kontrolle kommt, sind alle erstaunt, dass er es zu Hause *„so lange geschafft hat“.*

P ist weiter tumorfrei, hat aber zunehmende Zeichen der Unterernährung und einer chronischen Aspirationspneumonie. Einen Tag nach dem Kontrolltermin teilt die Ehefrau telefonisch mit, dass P zu Hause *„friedlich“* verstorben sei.

### Ethisch-rechtliche Betrachtung des Falles und Conclusio

Ärztliches Entscheiden (Festhalten am kurativen Ansatz) war beeinflusst von der Angst vor medicolegalen Konsequenzen *(„Patient tumorfrei!“). *Die Stimmung im Team war getragen von Frustration, dass P gut gemeinte Therapievorschläge ablehnte. Es war zu wenig klar, dass eine Behandlung nur stattfinden darf, wenn Patient:in diese auch wünscht. Auch ein fraglich entscheidungsfähiger Patient, der seine Wünsche klar äußern kann, und die Konsequenzen versteht (hier evtl. an Unterernährung/Aspirationspneumonie zu sterben), hat das Recht, eine Behandlung abzulehnen – Ärzt:in und medizinisches Personal soll diesen Weg nach Aufklärung und schriftlicher Dokumentation palliativ begleiten [67, 70, 71]. In Anerkennung des Selbstbestimmungsrechts unserer Patient:innen müssen wir aushalten lernen, dass diese manchmal einem gut gemeinten Rat nicht folgen wollen, um so die Ablehnung einer vorgeschlagenen therapeutischen Handlung nicht als persönliche Verletzung zu empfinden.

Erst durch Beiziehen der KEB konnte die Behandlung in eine von P gewünschte, die Ehefrau entlastende und auch für das Behandlungsteam akzeptable palliative Richtung gelenkt werden. Im Sinne der *relationalen Autonomie* musste neben P auch für seine Ehefrau ein unterstützendes Setting geschaffen werden (mobiles Palliativteam). In diesem vom P gewünschten und für die Frau *„sicheren“* Szenario lebte P dann palliativ begleitet – länger als erwartet – bis zu seinem Sterben zu Hause ein *gutes Leben am Ende des Lebens*. Wäre dem Behandlungsteam von Anfang an die ethisch/rechtliche Situation klarer gewesen, dann hätte sehr viel Stress für alle Beteiligten vermieden werden können.

Eine KEB kann dem Behandlungsteam einen ethisch und rechtlich untermauerten Handlungsvorschlag unterbreiten. Dies kann zur emotionalen Entlastung und Zufriedenheit trotz einer schwierigen Entscheidungssituation beitragen. Die Implementierung und systematische Förderung einer KEB ist daher eine notwendige Voraussetzung, um im Spannungsfeld zwischen Machbarkeit und Menschlichkeit eine Rückbesinnung auf Patient:innenwohl, Gerechtigkeit und Respekt vor der Autonomie zu ermöglichen. Wie andere ärztliche Kernkompetenzen auch, muss ethisch begründete Entscheidungsfindung gezielt gelehrt, erlernt und trainiert werden. Die Schulung von Studierenden und Ärzt:innen kann maßgeblich dazu beitragen, die Gefahr der Übertherapie und die Durchführung von *Non-Beneficial Treatments* zu reduzieren.

Nur so lässt sich eine Rückbesinnung auf eine menschliche und zugleich rationale Medizin erreichen, die sich nicht in technischer Machbarkeit verliert, sondern der Würde jedes einzelnen Menschen verpflichtet bleibt.

## Wie Klinische Ethikberatung Handlungs- und Rechtssicherheit in der Organisation Krankenhaus unterstützt (Stefan Dinges, Klara Doppler, Maria Kletečka-Pulker)

### Einleitung

Als Mitglieder im Leitungsteam der Klinischen Ethikberatung (KEB) AKH/MedUni Wien freuen wir uns, wie viel Resonanz und Bestätigung unsere Arbeit aus vielen Perspektiven/Reflexionen erhalten hat. Das erleichtert es, an dieser Stelle prioritär auf den organisatorischen und organisationalen Rahmen einer Ethikberatung einzugehen.

### Ethische Aufmerksamkeit im Alltag der Organisation Krankenhaus verankern

Die Gesundheitsberufe besitzen – neben ihrer fachlichen Expertise – eine hohe moralische und ethische Sensitivität, eine wesentliche und jahrhundertealte Motivation, in diesem Berufsfeld zu arbeiten: „that sensibility of heart which makes us feel for the distresses of our fellow creatures, and which, of consequence, incites us in the most powerful manner to relieve them“ [80].

Im Alltag der Expert:innenorganisation Krankenhaus gerät dieses humane Ethos der Medizin in vielfältige Spannungsfelder: Eine gewisse Konkurrenz herrscht zwischen den unterschiedlichen Fachlichkeiten, aber es stellt sich auch zunehmend unter dem Stichwort ‚Ethik und Monetik‘ die Frage nach der Wirtschaftlichkeit von Gesundheitseinrichtungen [81] – und keine Frage, auch die Dienstleistung KEB braucht ausreichend Ressourcen, um wirksam zu werden. Deswegen ist sicherzustellen, dass ethische Fragen nicht untergehen, eben keine störenden Fragen sind, sondern als Teil einer ethischen Versorgungsqualität verankert werden [82].

Dazu braucht es vereinbarte Routinen, in denen moralische Konflikte und ethische Dilemmata besprochen und bearbeitet werden. Gibt es diese Strukturen nicht, kann bei Mitgliedern der Gesundheitsberufe ein sog. moralisches Belastungserleben entstehen und zu ‚moral distress‘ führen [83, 84].

### Etablierte Strukturen von Ethikberatung sind ein Beitrag organisationaler Resilienz

Ethik, Wirtschaftlichkeit und hohe medizinisch-therapeutische Fachlichkeit sind kein Widerspruch, sondern in ihrem Zueinander die Bedingung der Möglichkeit, dass Menschen in schwerer Krankheit und am Lebensende in Krankenhäusern bestmöglich versorgt werden, ohne dass die Mitarbeiter:innen selbst den (moralischen) Halt verlieren oder selbst krank werden.

Das Allgemeine Krankenhaus Wien als Teil des Wiener Gesundheitsverbunds und die MedUni Wien haben sich aus gutem Grund dazu entschieden, Klinische Ethikberatung zu etablieren und zu nutzen. In Anlehnung an die Standards [85] der Fachgesellschaft Akademie für Ethik in der Medizin (AEM) steht zunächst das Angebot von Ethikfallberatungen im Mittelpunkt. Das wäre nicht möglich ohne kontinuierliche Aus- und Weiterbildung der Ethikkompetenzen, und damit der Förderung der ethischen Aufmerksamkeit aller Gesundheitsberufe. Die Klinische Ethik ist Aufgabe aller Gesundheitsberufe und Teil ihrer Expertise, nur ein kleinster Anteil der alltäglichen Handlungen und Entscheidungen benötigt KEB!

Neben der Erarbeitung von Ethikleitlinien ist die chronologisch letzte Aufgabe der KEB der Ethiktransfer: Die Ergebnisse aus einzelnen Ethikfallberatungen, der Ethikleitlinienarbeit und der Beratungen in klinikweiten Ethikboards fließen zurück in den klinischen Alltag und in die Klinische Ethik. Dafür sorgen insbesondere die Leitungsverantwortlichen, die KEB fördern und als Beitrag zur ethischen Resilienz nutzen [86].

Klinische Ethik ist Leitungsaufgabe und braucht deren ausdrücklichen Auftrag.

„Klinische Ethikberatung unterstützt und begleitet Klinische Teams in Wert- und Zielkonflikten in individuellen Behandlungs- und Versorgungsprozessen durch:

- *Strukturierte Moderation (Standortbestimmung – Zielfindung – Methodenwahl)*
- *Wertschätzende Kommunikation*
- *Beteiligung aller relevanten Perspektiven*
- *Ethische Begründungen und Empfehlungen*
- *Gemeinsam erarbeitete Ergebnisse in den jeweiligen Verantwortlichkeiten“ [*87*].*

Diese Auftragsklärung in der KEB ist in ihrer Methodenwahl, Rollenklärung und Haltung zugleich Ausdruck von Prozessqualität: KEB gestaltet Beratungsprozesse als Intervention, KEB interveniert in das Verhältnis eines klinischen Teams zu der bestmöglichen Praxis zugunsten eines/einer Patient:in, im Rahmen einer medizinisch-therapeutischen Indikationsstellung (die zugleich Auskunft über sinnvoll-erreichbare Ziele gibt) und der unbedingten Beachtung des Patient:innenwillens.

Um es zu verdeutlichen: KEB weiß zu Beginn einer Ethikfallberatung nicht, welches Ergebnis am Ende stehen wird – weil das Ergebnis nur in Beteiligung aller Betroffenen und deren Expertise im Respekt der Patient:innen-Autonomie erreicht werden kann.

Aber KEB sorgt durch ihre Prinzipien und Struktur dafür, dass ein moralisch-ethisch vertretbares Ergebnis möglich ist.

### Rechtssicherheit durch Klinische Ethikberatung

Die Voraussetzungen für jede medizinische Maßnahme sind die Einwilligung der Patient:in und die medizinische Indikation. Ist die Patient:in nicht entscheidungsfähig und besteht keine Gefahr im Verzug, bedarf es der Zustimmung einer Vertreter:in. Diese:r kann von der Patient:in im Voraus – also in entscheidungsfähigem Zustand – durch eine Vorsorgevollmacht [88] festgelegt worden sein. Weiters besteht die Möglichkeit, dass die Patient:in von ihrem Recht auf Selbstbestimmung Gebrauch gemacht hat, indem er/sie im Rahmen einer Patientenverfügung [67] bestimmte Maßnahmen antizipiert ablehnt. Dies lässt jedoch häufig dennoch Spielraum für andere Entscheidungen, bei denen es darum geht, den mutmaßlichen Willen der Patient:in bestmöglich zu ermitteln.

Hat die Patient:in rechtlich nicht vorgesorgt, sieht der Gesetzgeber verschiedene Vertretungsmöglichkeiten vor. Hier stellt sich bei Behandlungsentscheidungen oft die Frage, wie dem Willen der nun nicht mehr entscheidungsfähigen Patient:in bestmöglich Rechnung getragen werden kann. Besonders schwierig ist dies in der Praxis, wenn der/die Erwachsenenvertreter:in eine fremde Person ist – etwa ein:e Rechtsanwält:in, Notar:in oder ein:e Vertreter:in eines Erwachsenenschutzvereins.

Die Erfahrung von Jurist:innen in Ethikberatungen zeigt, dass insbesondere in Einrichtungen, die erst am Beginn der Etablierung von Ethikberatungen stehen, häufig versucht wird, im Rahmen der Beratung den mutmaßlichen Willen der Patient:in zu ermitteln. Dabei zeigt sich oftmals auch die Notwendigkeit, eine gesetzliche Vertreter:in zu bestellen, wenn dies bislang nicht erfolgt ist – etwa, weil zuvor nicht absehbar war, dass die Patient:in dauerhaft entscheidungsunfähig bleiben würde, oder weil man bisher mit der Regelung des „Gefahr im Verzug“ ausgekommen ist.

In Einrichtungen, in denen die Ethikberatung bereits länger etabliert ist, besteht meist ein breiteres Wissen über die juristischen Möglichkeiten und Notwendigkeiten. Jurist:innen werden dort vor allem in komplexeren Situationen hinzugezogen.

Neben der Ermittlung des Patient:innenwillens ist selbstverständlich auch die medizinische Indikation Gegenstand der Ethikberatung – oft sogar in enger Wechselwirkung, da beide Aspekte einander bedingen und beeinflussen. Dies gilt insbesondere dann, wenn mehrere Behandlungsoptionen zur Verfügung stehen.

Wie auch bei gemeinsamen Entscheidungen und Diskussionen in anderen Expertengremien – etwa in Kinder- und Opferschutzgruppen [89], Tumorboards oder Konsilen – schaffen gemeinsam getroffene Entscheidungen Rechtssicherheit. Denn es entscheidet nicht eine einzelne behandelnde Person, sondern es erfolgt eine gemeinsame Reflexion, bei der unterschiedliche Perspektiven der beteiligten Berufsgruppen sowie die Wahrnehmungen der Patient:in oder seiner/ihrer Angehörigen eine zentrale Rolle spielen.

### Lebensende und Sterben zulassen – eine permanente ethische Herausforderung

Im Blick auf unser Patientenbeispiel und die Erfahrung der Ethikberatung lässt sich zeigen: Zum einen – in Bezug auf die Tumorerkrankung – sehen wir ein erfolgreiches Team, zum anderen einen Patienten, der aufgrund der nicht mehr möglichen Rehabilitationsfähigkeit in Bezug auf Essen und Trinken an eine Grenze seines Lebens gestoßen ist und weitere medizinische Maßnahmen wie die Anlage einer Perkutanen Endoskopischen Gastrostomie (PEG)-Sonde ablehnt. Sein Wunsch ist es, nach Hause zu können, da er seine letzten Tage nicht im Krankenhaus verbringen möchte. Dieser Wunsch ist nicht nur eine medizinisch-organisatorische Herausforderung, sondern auch eine ethische: Mit dem Fokus auf Lebenserhaltung und dem Handlungsprinzip „in dubio pro vita“ fällt es schwer, das fünfte Handlungsziel und -feld, nämlich das „Sterben zulassen“ rechtzeitig zuzulassen – und damit im Sinne des Schweizer METAP-Modells [90] aus Sicht des Patientenwillens und Patientenwunsches, Über- und Unterversorgung zu vermeiden.

Im Prozess der KEB konnte hier das interprofessionelle Behandlungsteam einen Wechsel in der Therapiezielfindung gestalten und dem Patienten den Wunsch erfüllen, zu Hause mit seiner Frau auf dem Sofa zu sitzen und die mögliche Kost zu nützen. Den Preis und das Risiko von Unterernährung und Aspirationspneumonie wurde in Kauf genommen. An dieser Patienten- und Versorgungsgeschichte zeigt sich wiederum, dass am Lebensende, nach einer rehabilitativen Phase, die terminale Phase durchaus Tage und Wochen von Leben und Lebensqualität bedeuten kann – pro vita, in facie mortis – noch eine ganze Menge Leben, das dank medizinisch-therapeutischer Unterstützung und Expertise möglich war. Die finale Phase, der Sterbeprozess [91] und die letzten Stunden, waren eher kurz und nicht von Leid geprägt, und zu Hause.

## Wann ist Ethikfallberatung „gut“. Evaluation und Qualitätssicherung. (Jan Schildmann)

Unterschiedliche Perspektiven auf eine „gute Versorgung“ von Patient:innen sind, wie auch im geschilderten Fallbeispiel, häufig der Anlass für eine Ethikfallberatung. Es gilt dann herauszuarbeiten aus welchen ethischen Gründen eine Vorgehensweise als „gut“ beziehungsweise „gerechtfertigt“ angesehen werden kann. Während die Frage des „Guten“ oder „Richtigen“ in Bezug auf die Versorgung von Patient:innen Gegenstand zahlreicher Publikationen in der klinischen Ethik ist, liegen deutlich weniger Veröffentlichungen zum Thema „was ist eine gute Ethikfallberatung“ vor [92].

Während auf die Bedeutung einer angemessenen Evaluation von Ethikfallberatung in der Literatur seit langem hingewiesen wird [93–95], ist das Thema sowohl in der Forschung als auch mit Blick auf praxisorientierte Empfehlungen bislang eher wenig bearbeitet. Die Ursachen hierfür sind vielschichtig und reichen von pragmatischen Gründen, wie die vielerorts fehlenden Ressourcen für eine fundierte Evaluation, bis hin zu methodischen Herausforderungen einer angemessenen Evaluation.

Die Evaluation von Ethikfallberatung kann anhand von drei etablierten Typen von Qualitätsparametern erfolgen [96]. Die *Strukturqualität* bezieht sich auf die personellen, materiellen und organisatorischen Voraussetzungen der Ethikfallberatung. So könnte im Rahmen der normativen Evaluation der Strukturqualität eines Ethikfallberatungsangebotes bspw. geprüft werden, ob die Ethikberatenden angemessen ausgebildet wurden.

Die *Prozessqualität* umfasst Merkmale, mit denen Verfahrensweisen bei den verschiedenen Angeboten der Ethikfallberatung erfasst werden können. Die *Ergebnisqualität *bezieht sich zumeist auf Effekte von Angeboten der Ethikfallberatung auf die Patient:innenversorgung [97]. Ein Beispiel für die Evaluation der Ergebnisqualität ist die Prüfung, ob die Durchführung von Ethikfallberatung die Anzahl von Sterbenden erhöht – im Vergleich zu einer zufällig ausgewählten zweiten Gruppe von Patient:innen, bei denen keine Ethikfallberatung durchgeführt wird. Dieses Kriterium der Ergebnisqualität wurde in mehreren Studien zur Evaluation von Ethikfallberatung verwendet und zeigt gleichzeitig die Schwierigkeiten dieser Art der Evaluation auf. So konnten Larry Schneiderman und sein Team in der vielzitierten multizentrisch randomisierten Evaluation von Ethikfallberatung zeigen [5], dass die Sterblichkeit bei Durchführung von Ethikfallberatung auf der Intensivstation nicht signifikant ansteigt. Gleichzeitig wurden in der Interventionsgruppe mit Ethikfallberatung signifikant weniger intensivmedizinische Maßnahmen bei den Patient:innen durchgeführt, die auf der Intensivstation im Studienzeitraum verstarben. Diese auf den ersten Blick positiven Ergebnisse werfen aber Fragen nach den Zielen von Ethikfallberatung und inhaltlich angemessenen, gut messbaren Endpunkten auf. So ist bspw. zu fragen, ob ein Anstieg der Sterblichkeit ein negatives Ergebnis für die Qualität der Ethikfallberatung gewesen wäre. Die Entscheidung, das Sterben zuzulassen, ist schließlich ein häufiges und ethisch gut begründetes Ergebnis von Ethikfallberatung, wenn etwa der mutmaßliche Wille darauf hindeutet, dass die Patient:innen in der entsprechenden Situation keine weitere lebenserhaltende Behandlung gewollt hätten.

Die vorstehenden Ausführungen machen deutlich, dass eine angemessene Evaluation der Ergebnisqualität der Ethikfallberatung anspruchsvoll ist. Grund hierfür ist, dass Parameter identifiziert werden müssen, die einerseits das Ziel der Ethikfallberatung inhaltlich widerspiegeln und andererseits empirisch gut messbar sind.

Häufig werden aus der Gesundheitsforschung bekannte und scheinbar einfach zu messende Parameter verwendet, die bei genauerer Betrachtung allerdings Fragen hinsichtlich ihrer Angemessenheit für den Gegenstand der Ethikfallberatung oder anderer Angebote der Ethikberatung aufwerfen. Dies gilt neben den in der oben beschriebenen Schneiderman-Studie genannten Kriterien auch für das häufig verwendete Kriterium der Zufriedenheit. Informationen über die Zufriedenheit oder Unzufriedenheit der Teilnehmer:innen sind zweifellos ein wichtiger Hinweis für die Durchführenden von Ethikfallberatung. Allerdings sollte bedacht werden, dass das primäre Ziel der Ethikfallberatung nicht die Steigerung der Zufriedenheit, sondern eine ethisch gut begründete Entscheidung ist. Diese kann bei einem Teil der Beteiligten auch Unzufriedenheit auslösen, etwa dann, wenn die Wünsche von Angehörigen im Rahmen der Ethikfallberatung zwar geäußert werden können, aber nicht umgesetzt werden. Eine mögliche Alternative zur Messung der Zufriedenheit wäre die Messung der Akzeptanz der Beratungsergebnisse.

Ein letzter wichtiger Punkt, der bei der Evaluation von Ethikfallberatung beziehungsweise allgemein bei der Bestimmung der Qualität bedacht werden sollte, ist die Rückbindung von Qualitätsparametern an die Methode der Ethikfallberatung. Die geforderten Kompetenzen im Rahmen einer Ethikfallberatung können sich in Abhängigkeit vom zugrunde liegenden theoretischen und methodischen Ansatz der Ethikfallberatung unterscheiden. Hermeneutisch-tugendethische Ansätze gehen bspw. davon aus, dass die ethische Kompetenz der an der Ethikfallberatung teilnehmenden Personen sicherstellt, dass für das vorliegende ethische Problem angemessene Lösungsansätze im Sinne eines Konsens gefunden werden können.

Demgegenüber wären bei der Umsetzung der prinzipienorientierten ethischen Falldiskussion zu fordern, dass die Prozessschritte einer solchen Methode angemessen umgesetzt werden und die Entscheidung mit Hilfe der prinzipienorientierten Ethik begründet werden kann.

Zusammenfassend gilt: Die Tatsache, dass eine Ethikfallberatung stattfindet, ist kein Garant für eine ethisch gut begründete Entscheidung in der Patient:innenversorgung. Die begründete Auswahl einer Methode der Ethikfallberatung und deren kompetente Anwendung sind hierfür notwendig. Bei der Evaluation von Ethikfallberatung müssen neben Anforderungen, die in der Methode der Ethikfallberatung begründet sind, auch die Messbarkeit von Qualitätsparametern sowie nicht zuletzt die zur Verfügung stehenden Ressourcen bedacht werden. Angesichts der weitreichenden Auswirkungen von Ethikfallberatung in der Patient:innenversorgung, wie dies auch im vorgestellten Fallbeispiel deutlich wird, sind Evaluationsstudien, nicht zuletzt für das Vertrauen in das Angebot, von großer Bedeutung [98].

## Nachwort zu „Lieben Sie Ethik“ – Symposium zur klinischen Ethikberatung am 25. November 2024 (Christiane Druml)

„*Bioethical pioneers at the frontier of human history, we affirm that the past does not inexorably determine the future – and that it is precisely through this historic effort to explore and promote values in the world for which we share responsibility, that we express confidence in our own lives, (…) and in the future of our world*.“

Dieser Ausspruch ist Jonathan Mann zugeschrieben. Er war einer der Pioniere auf dem Gebiet von Health and Human Rights, Professor an der Harvard School for Public Health und hat UNAIDS gegründet. Leider ist er 1998 viel zu früh bei einem Flugzeugabsturz vor der Kanadischen Westküste ums Leben gekommen.

Pioniere gibt es auf den verschiedensten Gebieten und gerade die Ethik braucht sie! Klinische Ethikberatung ist die „Dritte Kompetenz“ neben den als ersten in Österreich etablierten (Forschungs‑) Ethikkommissionen, die es seit den 1970er-Jahren an der medizinischen Fakultät/dem Allgemeinen Krankenhaus in Wien und später in ganz Österreich gibt und neben der österreichischen nationalen Bioethikkommission beim Bundeskanzleramt, die 2001 vom damaligen Bundeskanzler gegründet wurde.

Ethikkommissionen befassen sich mit klinischer Forschung und beurteilen Protokolle und ob die Integrität der in diese Projekte einbezogenen Menschen gewahrt bleibt. Ohne sie kann kein Forschungsprojekt durchgeführt und die Ergebnisse publiziert werden. Bioethikkommissionen hingegen beraten eine Regierung oder das Parlament in Bezug auf gesellschaftliche Folgen, die die Entwicklung der Lebenswissenschaften mit sich bringen. Ein klassisches Thema einer Bioethikkommission ist die embryonale Stammzellforschung bzw. heute Embryomodelle und alles, was mit dem Beginn oder dem Ende des Lebens zu tun hat.

Einige weitere Jahre hat es dann gedauert, bis 2024 durch „Pioniere“ von Seiten der Palliativmedizin und der Intensivmedizin an der seit 2004 eigenständigen Medizinischen Universität Wien eine klinische Ethikberatung, die dritte Kompetenz, ins Leben gerufen wurde. Die Klinische Ethikberatung ist in einer Zeit wie der heutigen, in der die Moral zunehmend in der Öffentlichkeit eingefordert wird, sehr gefragt und benötigt eindeutig fachliche Kompetenz.

Klinische Ethikkomitees beziehen sich auf den einzelnen Menschen, auf das Individuum und seine Behandlung. Hier geht es um schwerwiegende Fragen, ob eine Therapie weitergeführt werden soll oder nicht, oder ein Organ transplantiert. Auch diese Komitees sind interdisziplinär zusammengesetzt und müssen Entscheidungen treffen, wiewohl die behandelnden Ärztinnen und Ärzte nach wie vor die Letztverantwortung tragen.

Wenn das behandelnde Team einig ist über eine medizinische Entscheidung, dann benötigt es keine externe Beratung und Begleitung seiner Handlungen. Dennoch kann eine qualitativ hochwertige ergebnisoffene Beratung und Moderation einer Entscheidungsfindung in schweren Situationen wertvoll sein und den Akteuren Rückhalt geben. Vielleicht gerade dann, wenn Familien andere Wünsche haben als die Patientin oder der Patient. Sie können auch helfen, der Falle der Übertherapie zu entgehen.

Klinische Ethikkomitees können zwar den behandelnden Ärztinnen und Ärzten nicht die moralische Verantwortung abnehmen, durch ihre Beratung aber unterstützen und eine neue Qualität in die Entscheidungen bringen.
